# Calibration of the Pulse Signal Decay Effect of Full-Waveform Hyperspectral LiDAR

**DOI:** 10.3390/s19235263

**Published:** 2019-11-29

**Authors:** Changsai Zhang, Shuai Gao, Zheng Niu, Jie Pei, Kaiyi Bi, Gang Sun

**Affiliations:** 1 The State Key Laboratory of Remote Sensing Science, Aerospace Information Research Institute, Chinese Academy of Sciences, Beijing 100101, China; zhangcs@radi.ac.cn (C.Z.);; 2 University of Chinese Academy of Sciences, Beijing 100049, China

**Keywords:** hyperspectral LiDAR, range ambiguity, point clouds, pulse decay correction, vegetation

## Abstract

Full-waveform hyperspectral LiDAR (FWHSL) is able to obtain spectral and spatial information by utilizing a single instrument, and it has become more and more commonly used in vertical distribution studies of structural and biochemical characteristics of vegetation. However, the pulse-echo arrival times of multiple spectral channels of the FWHSL are not consistent and this causes range ambiguity in spectral channels. In this paper, the pulse signal decay effect on range measurements was studied by measuring the varying trends of pulse signal decay between spectral channels with different material properties. The experiments were repeated at different distances. All of the spectral channels were compared for different materials. The results suggest that the channels in the red edge spectral region of vegetation have good stability and accuracy for range measurements of varied distance and materials properties. Finally, based on the geometric invariability in a specific red edge channel, a practical calibration approach for the pulse signal decay effect is also presented. The validation tests showed it could improve the pulse signal decay effect of full-waveform hyperspectral LiDAR.

## 1. Introduction

Full-waveform multi/hyper-spectral LiDAR (FWHSL) has recently been adopted as a valuable survey tool to reduce the cost of field measurements, accurately characterize vegetation structure, and estimate leaf and canopy biochemical contents [1,2,3,4,5,6,7,8,9,10,11,12]. In particular, the advent of the nonlinear fiber optics and full-waveform LiDAR technology [13], which is capable of recording entire reflected pulse waveforms of multiple spectral channels from visible to near-infrared spectral region, has enabled this advantage to become more conspicuous. Within a forest environment, the broadband directional light of the supercontinuum laser source could penetrate dense canopies through small gaps and thus provide a time-versus-spectrum profile by post-processing the recorded waveforms of multiple channels. Consequently, more detailed spatial distributions of biophysical and biochemical parameters can be revealed through these data. However, the FWHSL laser pulse-echo signals of different spectral channels for the same observation target have different pulse-echo arrival times, complicating the use of direct applications of range values to infer geometric information of a vegetation structure. There is a duplicated imaging effect in 3D point clouds data due to the different range values of multiple spectral channels.

For data from an observation target acquired by the FWHSL system, the difference in pulse-echo arrival time between spectral channels is mainly caused by instrumental characteristics—that is, the effects of group-velocity dispersion and nonlinear effects of the supercontinuum laser source, the nonlinear effect of optical pulse propagation in the instrument [14,15,16,17], and the response of the multichannel photodetector—given that atmospheric attenuation is negligible. Asynchronization has been shown to occur between different spectral channels of the FWHSL. Such asynchronization is mainly caused by the inherent characteristics of the supercontinuum laser source, indicating that each wavelength is not strictly synchronized (i.e., they have a slightly different timing at the beginning of transmission) [18,19]. With an increase in wavelength, the laser pulse emission time occurred earlier, and the maximum difference time of the pulse is hundreds of picoseconds. Moreover, a saturation of the photodetector could also affect the apparent rise time of the detected pulse (by depressing the peak level) and thus introduce an intensity-dependent bias in the measurement of the return time. In addition, the fixed length of optical path inside the instrument and the random quantization error also affect pulse delay results. Finally, the pulse signal decay effect causes range ambiguity in multiple spectral channels. Therefore, eliminating the effects of the pulse-echo time difference between spectral channels is essential for making effective use of the geometric range data of FWHSL.

In this study, we develop a laboratory-based prototype of a full-waveform hyperspectral LiDAR [20] designed for vegetation applications. This system is designed to take measurements in 20 channels (from 540 to 849 nm, with a spectral sampling interval of 17 nm). Our study used data from this FWHSL system. The objective of this study is to eliminate the pulse signal decay effect on range measurements of the FWHSL. Experiments were conducted to evaluate varying trends of pulse-echo arrival time between spectral channels.

## 2. Related Work

The existing hyperspectral LiDAR system has a wide emission spectrum from a supercontinuum laser source (SC) and a photodetector or multi-channel detector for receiving backscattered echo signals. The current system has fewer than 32 available spectral channels with high signal-to-noise ratio. The HSL is designed for vegetation detection [21]. A laser beam separated by L2 is focused on an avalanche photo diode (APD) detector and then gathered into the acquisition system, which is used to trigger a measurement and to capture the echo. The echo signals are guided into a grating spectrometer which is used for separating light echoes and then be converted to an electrical signal by a photosensitive array. The AOTF-HSL is designed to provide data for calculating the vegetation indexes in vegetation remote sensing applications [22]. The broadband output laser beam is filtered firstly by the acousto-optic tunable filter (AOTF) device, which is a wavelength selection module based on the acousto-optic crystal, and then focus the echo signals directly on the photodetector installed on the focal plane of the telescope. The full waveform HL is designed for vegetation remote sensing and 3D identification [23]. The broadband output laser beam passes through a beam sampler, which takes a part of the beam for triggering the time-of-flight measurement. An off-axis parabolic mirror is used as the primary collecting optic and an APD array module is used to convert the spectrally separated light to analog voltages. The LCTF-HSL is designed to detect the red edge for vegetation-related application [24]. The SC laser beam passes through a beam sampler, which utilizes a minor part of the collimated beam for triggering the time-of-flight measurement. A liquid crystal tunable filter (LCTF) device is installed before the APD detector to select the appropriate wavelength band, which is a technique of time division multiple spectral filters.

**Figure 1:**
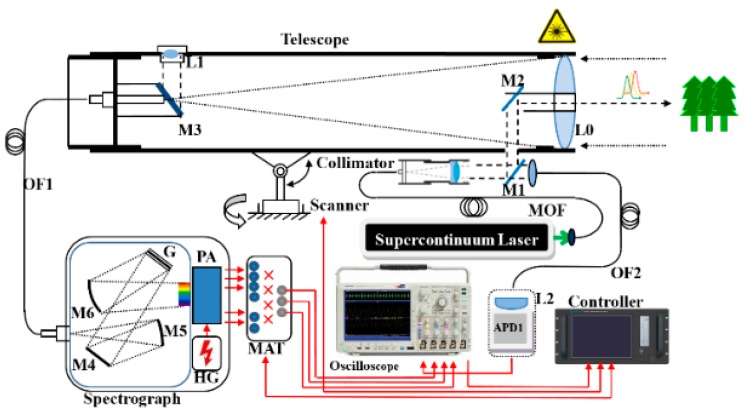
The optical layout of the employed full-waveform hyperspectral LiDAR (FWHSL) system.

During data acquisition, a trigger condition must be set before the data acquisition card can collect pulse signals and transmit them to the system in order to avoid unnecessary acquisition and storage of pulse signals. Two types of pulse trigger are possible: the first kind of trigger is channel specific and the second kind of trigger is universal total triggers. The most important purpose of taking specific channels as triggers is to accurately record the transmitted laser power of each wavelength of the LiDAR so as to perform radiation calibration based on the radar equation and more conveniently acquire the reflectance of target in each wavelength channel. However, the latter type is used for most existing instrument setups, chiefly because the receiving echo laser power would not be diverted significantly by the trigger channel. Meanwhile, the latter type cannot avoid the non-synchronization of emission time of different wavelength in SC laser source. Usually an optical filter [25] or a grating (see Figure 1) is installed before an APD or PMT and utilized as a spectroscopic device to select the wavelength of the backscattered echoes. Some spectroscopic devices can consist of an acousto-optic tunable filter or a liquid crystal tunable filter. AOTF and LCTF can cause more attenuation of the laser beam and more loss of outgoing laser power compared with optical filter and grating. However, AOTF and LCTF are the technique of time division multiple spectral filters, which alleviate the pulse delay effect caused by the SC laser source.

The pulse delay is a common phenomenon for hyperspectral LiDAR systems. Many of the hyperspectral instruments use different setups and the delay effect of different instruments is not exactly the same. The delay effect is mainly caused by four factors: supercontinuum laser source, photodetector, internal optical path, and random error. In practice, hardware processing can only alleviate the pulse delay effect, and it cannot completely solve the situation of different delay inconsistencies in different channels. On a hardware level, it is difficult to ensure that the range values of different channels of the same target are identical.

## 3. Materials and Methods

### 3.1. FWHSL System

We assembled an FWHSL system based on existing studies on the estimation of vegetation biochemical contents [19,20,26]. The optical setup for measuring the time-of-flight and return intensity of a broadband output laser pulse is shown in Figure 1. The FWHSL consists of a supercontinuum pulsed laser source, a coaxial optical transmitting and receiving telescope, a two-axis scanner, a spectrograph, and a data acquisition unit.

The supercontinuum laser (NKT Photonics, average output power of 100 mW, pulse repetition rate of 24 kHz, 2 ns pulse width, and broadband spectrum white light of ~450–2400 nm) produces a collimated laser beam with ~3 mrad divergence angle and ~4 mm beam diameter at output. A small portion of the emitted light beam passes through a semi-transmitting reflecting mirror M1, which transmits part of the beam through an optical fiber (OF2) to the avalanche photon diode (APD1) sensor. The APD1 sensor is used to collect a sample of the emitted pulse waveform, which triggers a measurement for capturing the emitted and echo waveforms. An achromatic refractor telescope (with a 400 mm focal length, and 80 mm aperture diameter) collected the scattered laser beam from the target. The backscattered beam is focused with the achromatic lens onto the grating spectrograph (with full width at half maximum about 10 nm for each channel and a spectral sampling interval of 17 nm), and divided into multiple channels for photoelectric detection. A linear array multianode photomultiplier (PMT) assembly is used to convert the spectrally separated light to analog voltages. The output signals from the PMT module are sampled and recorded using a high-speed oscilloscope (Tektronix DPO5024B, with 2 GHz bandwidth, 5 GS/s sampling rate for four analog channels). An average of 16 pulses is saved to improve the signal to noise ratio and to reduce the amount of data.

Due to the combination of the transmitted spectrum and the PMT array sensitivity, the effective spectral range of the FWHSL measurement is limited to between 540 and 849 nm. There are 10 visible channels at the wavelength before the red edge of the vegetation and 10 near-infrared channels, which enables the calculation of various vegetation indices.

### 3.2. Experimental Setup

**Figure 2:**
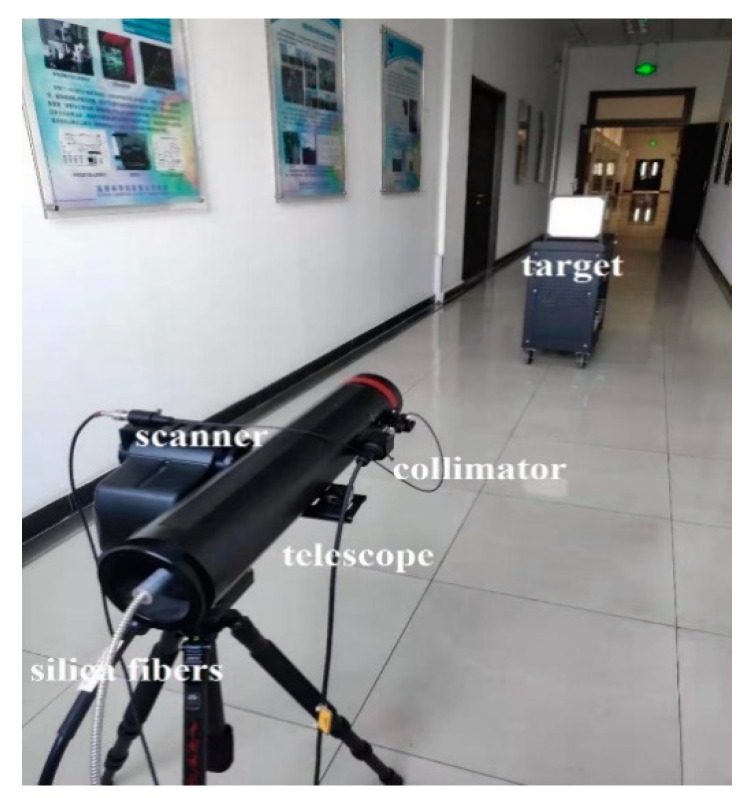
The FWHSL scanner hardware prototype.

Based on the schematic diagram described in Figure 1, the FWHSL system prototype was set up in the laboratory for pulse signal decay evaluation (see Figure 2). The laboratory experiments evaluated the peak pulse-echo time difference between spectral channels of the proposed FWHSL. This was done by estimating the relationship between the pulse echo time and material properties at various distances.

To study the effects of range distance and reflectance characteristic on the pulse-echo arrival time difference, three types of greyboards with nominal reflectance values of 30%, 40%, 50%, and a whiteboard (99%) were scanned at a position 4 m from the aperture of the telescope along the optical axis of the telescope. They were then moved away from the telescope at a step interval of 2 m until the distance reached 20 m.

**Figure 3:**
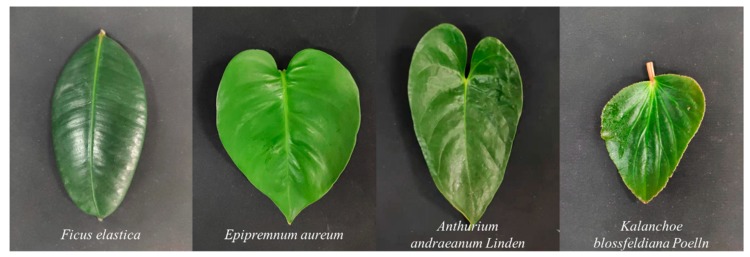
Measurement targets plotted on a smooth black surface.

**Figure 4:**
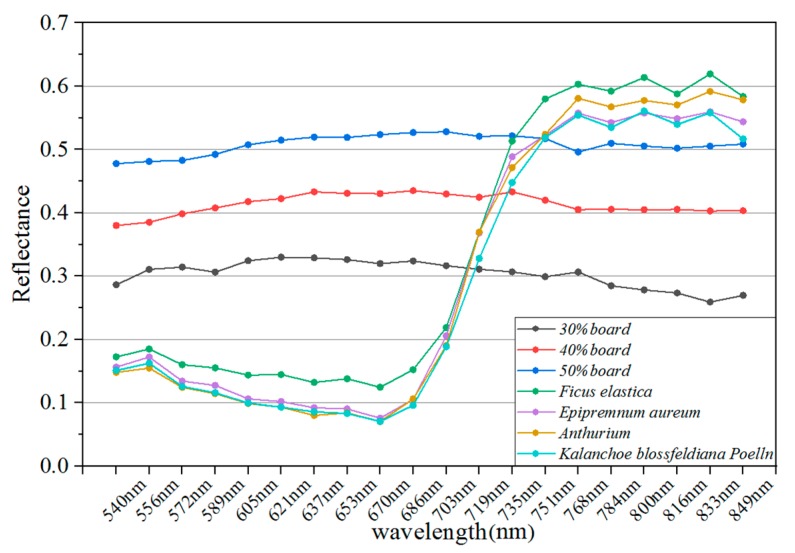
Spectral reflectance of the measured targets collected by FWHSL.

To estimate the pulse-echo arrival time difference for various material properties (spectral backscattering characteristics), four leaf samples were randomly collected from four broadleaf plant species and measured at different distances. Just shown in Figure 3, leaf species include *Ficus elastica* (denoted as plant leaf 1), *Epipremnum aureum* (plant leaf 2), *Anthurium andraeanum Linden* (plant leaf 3) and *Kalanchoe blossfeldiana Poelln* (plant leaf 4), and corresponding spectral reflectance collected by FWHSL were shown in Figure 4.

**Figure 5:**
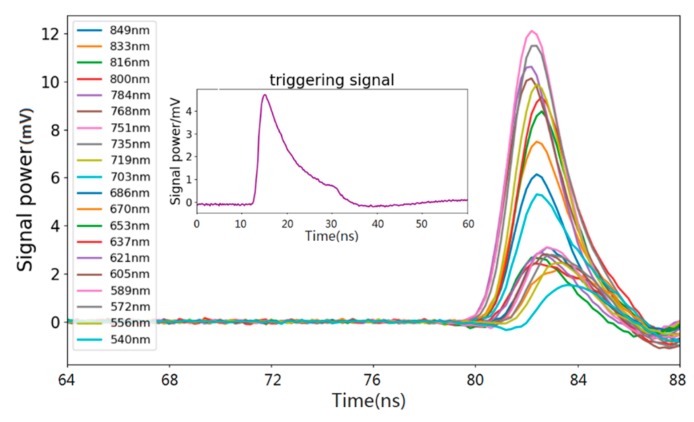
Spectral waveforms of a grey board collected by FWHSL.

For each reference target, one position was selected and measured at different distances, the average of five pulses was applied to improve the signal to noise ratio and to avoid signal miss as might occur with a single measurement. Spectral waveforms of a 50% greyboard collected by FWHSL at 6 m were shown in Figure 5. The pulse waveforms were fitted using a Gaussian model in order to catch the peak time position of the waveform, which might be missed due to the instrumental sampling. Meanwhile, a laser rangefinder (Leica, DISTO D5) was situated on the aperture of the telescope of the FWHSL and used to measure the reference distance value for the experiments.

The FWHSL primary distance measurement value based on the time-of-flight method includes two parts: (1) the distance from the telescope receiver to the reference target; and (2) the fixed light path length of the instrument (involving OF2, the telescope, and the spectrograph). The latter cannot be directly measured by the reference laser rangefinder.

To estimate range accuracy and pulse signal decay of the spectral channels, we define a range deviation as the relative difference between the primary distance and the actual reference distance. This is the sum of the fixed optical path length of the instrument and the inherent pulse signal decay range deviation of each channel.

## 4. Results and Discussion

**Figure 6:**
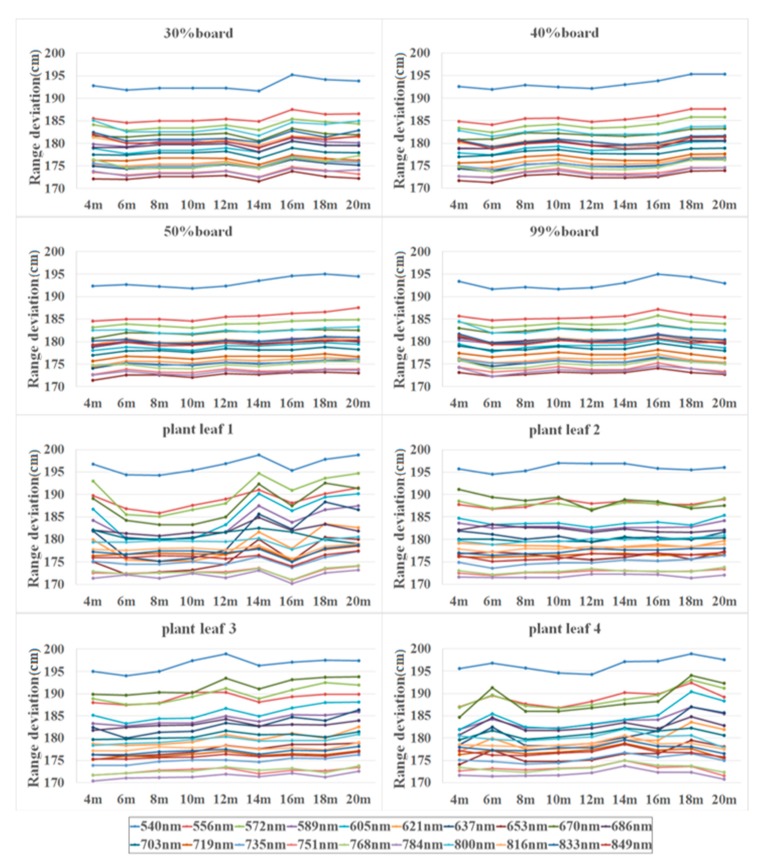
The range deviations of 20 channels generated from observations with eight reference targets in varying distances.

The following subsections describe results generated from experiments based on the FWHSL system. Figure 6 presents the range deviation of 20 spectral channels over various distances applied to eight different targets. The range deviation of each channel was different due to the pulse signal decay effect.

**Figure 7:**
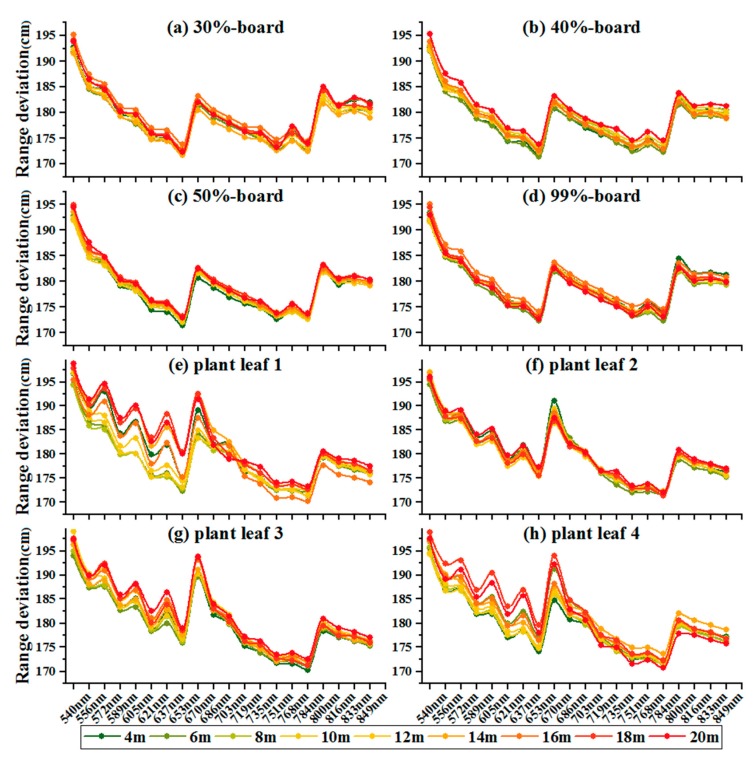
The range deviation spectra of eight reference targets in 20 channels.

**Figure 8:**
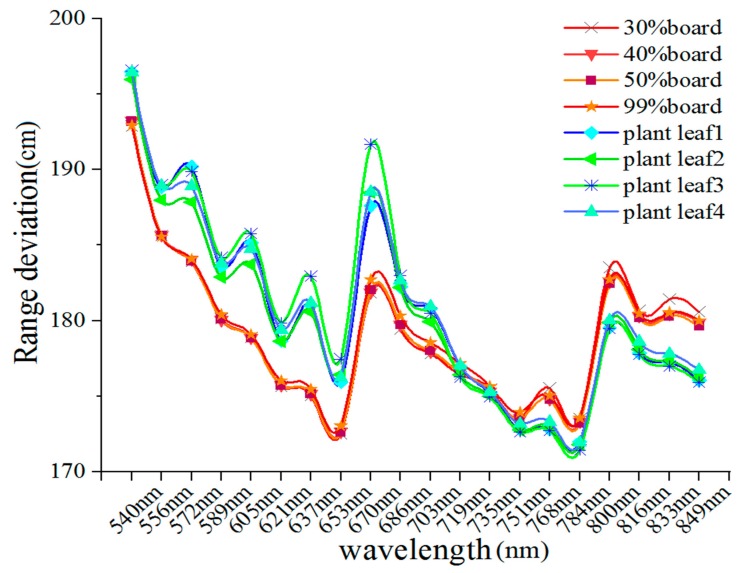
The range deviation distribution of eight reference targets in 20 channels.

To better understand the distribution of the range deviation of the 20 spectral channels, Figure 7 presents range deviation spectra at various distances. Each target exhibited a distinct response in different spectral channels. The range deviations of each target are distributed with a ‘W’ shape that was higher for shorter wavelengths than for longer wavelengths, especially for plant leaf samples. The following results can be observed from the range deviations conveyed by Figure 7 and Figure 8:

(1) The range deviations for the various spectral channels have obvious differences. The maximum difference of range deviation between spectral channels was approximately 21 cm;
(2) The overall trend of the deviation distribution was that the deviation decreases with increasing wavelength, which means that the pulse delay time decreases with the increasing wavelength;
(3) For four reference board targets, as increased distance, the range deviations of each channel were consistent over varying distances. The range deviations of each channel showed no correlation with distance;
(4) For four plant leaves, the range deviations of the visible wavelength channels exhibited small fluctuation over varying distances compared with those of the reference board targets.

The observed differences between the measured and actual distances in different wavelength channels can be explained as follows:

(1) The inherent characteristics of the supercontinuum laser source caused each wavelength to be not strictly synchronized because each one has a slightly different timing at the beginning of transmission. The temporal as well as spectral evolution of optical pulses, launched inside a highly nonlinear fiber of the supercontinuum laser source, is affected not only by a multitude of nonlinear effects, but also by the dispersive properties of the fiber [19]. Asynchronization was shown between different wavelength channels of the FWHSL. With increasing wavelength, the echo arrival time occurred earlier. Moreover, with increasing fiber length, the pulse signal decay time became longer.
(2) The supercontinuum laser source produces a collimated broadband spectrum laser beam and the outgoing pulse is collected by the APD detector using a universal total trigger (see Figure 1 and Figure 5). The spectral response range of the APD is 300–1000 nm, with a typical max responsivity at 730 nm. The peak wavelength of quantum conversion efficiency of the APD photo-detector is ~730 nm, which corresponds to the red edge spectral region of vegetation. This configuration implies that the range values of the red edge channels calculated by time-of-flight measurement method are the most accurate and stable.
(3) A possible source of nonlinearity is the response of the multichannel photomultiplier used for detection. A saturation of the detector could for instance affect the apparent rise time of the detected pulse (by depressing the peak level) and thus introduce an intensity-dependent bias in the measurement of the return time. In addition, crosstalk effects (i.e., spurious signal generated on one channel by the signal detected in another channel) could affect the output pulse shapes. These effects are not uncommon in multichannel photomultipliers, and even a small amount of nonlinearity in the response could generate the small deviations in delay time.
(4) The FWHSL primary distance measurement value based on the time-of-flight method includes two parts: the distance from the telescope receiver to the target and the fixed length of optical path inside the instrument after triggering the pulse emission signal. The latter caused an obvious bias between the measured and actual distance.
(5) The range deviation of each channel varied slightly with range distance due to the random quantization error of the FWHSL. The sampling rate of the oscilloscope was 5 G/s with an interval of 0.2 ns; therefore, the corresponding digitized random measurement error was 3 cm. Moreover, the flight time was calculated based on waveforms stored within the oscilloscope, which might have errors in calculating the interval time.

Figure 8 presents a comparison between the average range deviations of the nine measured distances. The distribution of the range deviation of the 20 channels resulting from the pulse signal decay effect is nonlinear and irregular for different material properties; it cannot be fitted directly by any mathematical function to correct the range deviations. However, the range deviation of the wavelength channels at the red edge position of the vegetation (719 nm and 735 nm) is consistent across all of the different material properties.

### Calibration of the Pulse Signal Decay

Spectral 3-D point cloud data (x, y, z, R(λ)) can be generated using hyperspectral LiDAR scanning. However, a distance bias between the spectral channels generated multiple (x_λ_, y_λ_, z_λ_, R(λ)) for the same measured target. As we can see in Figure 7 and Figure 8, the distribution of pulse signal decay times between spectral channels of the FWHSL was irregular except the channels at the red edge region. The range deviation of the channels in the red edge region did not vary with range distance, reflectance, or spectral backscatter characteristics. The range value of red-edge spectral channels showed good stability and accuracy for various materials properties.

In practice, the specific range value should be confirmed in order to avoid range ambiguity between the spectral channels caused by the pulse signal decay effect. For millimeter-thickness plant leaf blades, even a one-centimeter inconsistency between different channels is not acceptable. To calibrate the pulse signal decay effect, the range value of any of the channels at the red edge position of the spectral region of vegetation was selected as the unique range value *d*. Then, the unique range value subtracts the corresponding range deviation of the channel in the red edge in order to eliminate the deviation caused from the fixed length of optical path inside the instrument and the inherent pulse signal decay of the channel.

$$d=\frac{c\cdot (t_{end,\lambda_{719}}-t_{start})}{2}-d_{0}$$

where, *t_start_* is the start time of trigger pulse; *t_end_* is the peak time of echo pulse; *c* is the speed of light in vacuum. The calibrated range value *d* is consistent with the distance from the telescope receiver to the target and is used as the unique value to substitute for all 20 channel range values. By establishing a LiDAR calibration procedure based on geometric invariability in one specific channel, hyperspectral LiDAR offers the advantage of eliminating the duplicated imaging effect for the same observation target and avoiding range ambiguity in targets with different spectral backscatter characteristics.

**Figure 9:**
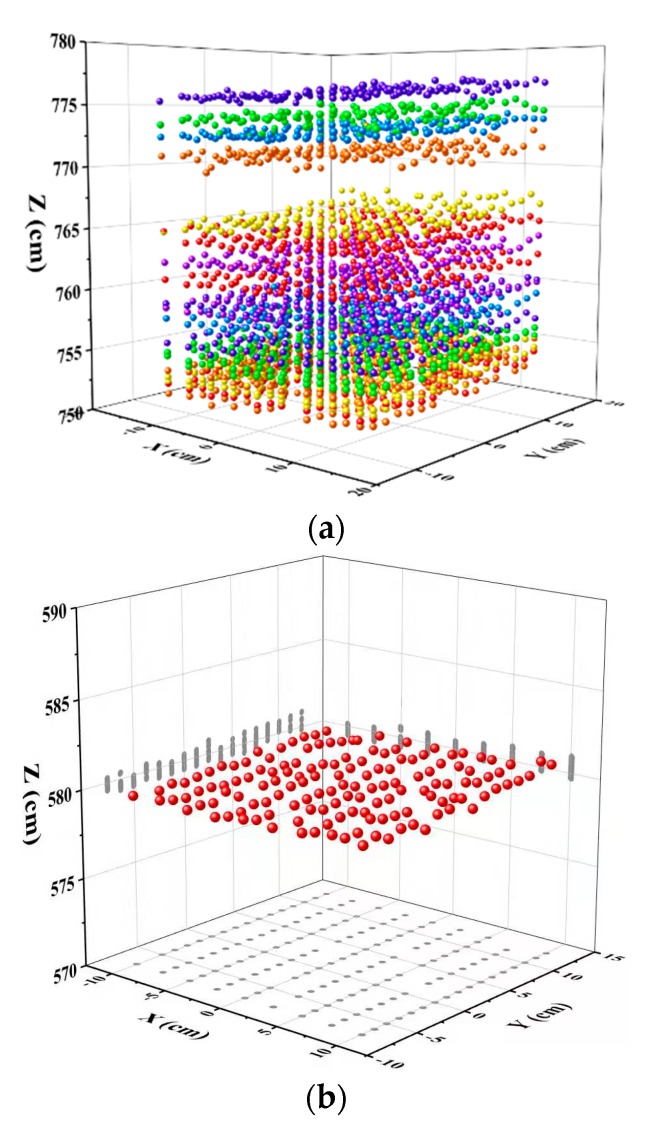
FWHSL pulse signal decay effect visualization; (**a**) before correction, (**b**) after correction.

Figure 9 shows 3D point cloud visualization of a reference board. The reference board with a size of 20 cm × 20 cm was scanned by the FWHSL sensor at a position of 5.8 m from the aperture of the telescope. There is a point cloud duplicated imaging effect for the same observation object due to the different range values of 20 channels (see Figure 9a). The advantage of the practical approach for correcting the effect of pulse signal decay is shown in Figure 9b, the calibrated result shown in Figure 9b is based on a specific channel (719 nm). After a calibration of the pulse signal decay effect, the point cloud duplicated imaging effect was eliminated and accurate three-dimensional geometric information of the reference board was obtained.

**Figure 10:**
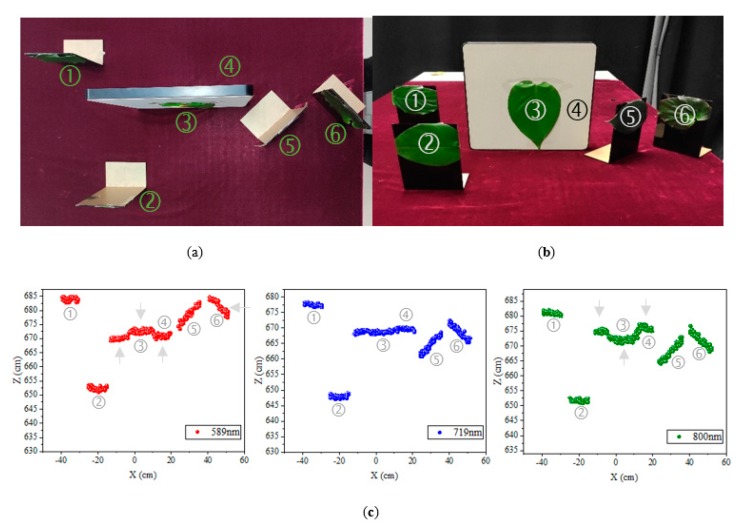
The indoor experimental scene with six materials; (**a**) Top view, and (**b**) Front view. (**c**) hyperspectral LiDAR point clouds presented in false color.

Figure 10 presents an indoor experimental scene with six materials. The experimental scene included various kinds of materials: Five different plant leaves (Targets 1~3 and 5, 6) and a grey board (Target 4). The vegetation and grey board materials were placed to validate the range measurement ability of the specific wavelength channel of the hyperspectral LiDAR. There was an interval distance of 30 cm between Target 1 and Target 2, 20 cm between Target 2 and Target 3. Target 3 was attached to the surface of Target 4. In addition, the targets with different angles of inclination were included.

The point clouds captured by the hyperspectral LiDAR are depicted as false color point clouds in Figure 10c. The 589 nm, 719 nm, and 800 nm channels of false color were assigned by red, blue and green, respectively. The results showed that the relative position of Target 4 moves forward in 589 nm channel and backward in 800 nm channel. In addition, the relative position of Targets 5 and 6 in 589 nm channel were obviously backward. For 589 nm and 800 nm channels, there were obvious deviations in the positions indicated by the gray arrow, which correspond to the deviations in the Figure 8. The advantage of unique channel was shown in 719 nm channel due to the geometric invariability in the red edge channel. Relative positions and shapes of all targets were well reconstructed by the point clouds collected by the hyperspectral LiDAR.

## 5. Conclusions

In this paper, we discussed the effects of pulse signal decay on FWHSL range measurements and proposed a practical calibration approach. The experiment results showed that the decay time of each channel varied with spectral backscattering characteristics, independent of measured distance and reflectance. The red-edge spectral channel was insensitive to object factors and advantageous in calibrating the effect of pulse decay. The relatively accurate three-dimensional geometric information of the target was obtained by using the calibrated range value. The proposed approach not only eliminated the duplicated imaging effect for the same observation target, but would also avoid range ambiguity in targets with different spectral backscatter characteristics in forest environments.

Hardware processing can alleviate the pulse delay effect but is unable to completely solve the situation of different channels delay inconsistencies. In this paper, the pulse delay effect is eliminated by an effective post-processing procedure. A calibration approach based on geometric invariability in a specific channel improves the calibration procedure of hyperspectral LiDAR during post-processing (i.e., on the software level). Overall, this study can be regarded as a practical solution to the calibration problem for the full-waveform hyperspectral LiDAR. However, with future development of the hyperspectral LiDAR system, the proposed approach should be further tested for various optical setups and additional calibration factors should be considered.
